# Unconditioned- and Conditioned- Stimuli Induce Differential Memory Reconsolidation and β-AR-Dependent CREB Activation

**DOI:** 10.3389/fncir.2017.00053

**Published:** 2017-08-10

**Authors:** Bing Huang, Huiwen Zhu, Yiming Zhou, Xing Liu, Lan Ma

**Affiliations:** The State Key Laboratory of Medical Neurobiology, School of Basic Medical Sciences and the Institutes of Brain Science, and Department of Translational Neuroscience, Shanghai Pudong Hospital, Fudan University Shanghai, China

**Keywords:** memory reconsolidation, unconditioned stimulus, conditioned stimulus, β-adrenergic receptor, pCREB

## Abstract

Consolidated long-term fear memories become labile and reconsolidated upon retrieval by the presentation of conditioned stimulus (CS) or unconditioned stimulus (US). Whether CS-retrieval or US-retrieval will trigger different memory reconsolidation processes is unknown. In this study, we introduced a sequential fear conditioning paradigm in which footshock (FS) was paired with two distinct sounds (CS-A and CS-B). The treatment with propranolol, a β-adrenergic receptor (β-AR) antagonist, after US (FS)-retrieval impaired freezing behavior evoked by either CS-A or CS-B. Betaxolol, a selective β1-AR antagonist, showed similar effects. However, propranolol treatment after retrieval by one CS (e.g., CS-A) only inhibited freezing behavior evoked by the same CS (i.e., CS-A), not the other CS (CS-B). These data suggest that β-AR is critically involved in reconsolidation of fear memory triggered by US- and CS-retrieval, whereas β-AR blockade after US-retrieval disrupts more CS-US associations than CS-retrieval does. Furthermore, significant CREB activation in almost the whole amygdala and hippocampus was observed after US-retrieval, but CS-retrieval only stimulated CREB activation in the lateral amygdala and the CA3 of hippocampus. In addition, propranolol treatment suppressed memory retrieval-induced CREB activation. These data indicate that US-retrieval activates more memory traces than CS-retrieval does, leading to memory reconsolidation of more CS-US associations.

## Introduction

In Pavlovian threat (fear) conditioning, an initially neutral conditioned stimulus (CS), such as a tone, is paired with an aversive unconditioned stimulus (US), typically an electric shock, which evokes pain or strong somatic discomfort (Maren, 2001b; Nader and Hardt, 2009). After a single pairing, the initially neutral stimulus exposure will elicit a spectrum of fear-like or defensive responses, like freezing (Rosen, 2004). Fear conditioning is a valuable tool for studying the neurobiological nature of associative memory and renders a way to explore organization of memory formation.

Memories were originally assumed to be static and inflexible through a consolidation process that stabilizes and stores the information acquired. More recently, evidence has emerged that memories are, in fact, dynamic and modifiable. Retrieval can result in synaptic destabilization (Lee and Everitt, 2008; Kim et al., 2010), and ensuing restabilization process, known as reconsolidation, involved *de novo* protein synthesis (Nader et al., 2000) and synaptic plasticity (Clem and Huganir, 2010). Thus, manipulation of reconsolidation process allows for memory modification and even memory elimination. In addition, consolidated memory may also undergo extinction with repeated CS induced memory retrieval, leading to an amenable process (Myers and Davis, 2002). Reconsolidation is now being studied extensively with CS-retrieval. Pharmacological or behavioral interventions following CS-retrieval can, like post-training manipulations, disrupt the long-term memory (LTM) of a variety of learning paradigms (Nadel and Land, 2000; Nader and Hardt, 2009; Reichelt and Lee, 2013). Recent studies reported that US presentation alone after fear memory consolidation also induced a reconsolidation process which, when disrupted by protein synthesis inhibitor, results in a decrease in CS-evoked behavioral fear memory in an amygdala-dependent manner (Debiec et al., 2010; Díaz-Mataix et al., 2011). The US presentation followed by extinction training impaired reinstatement of fear memory or drug memory in both humans and rats (Liu et al., 2014; Luo et al., 2015). So memory reconsolidation can be induced by US, as well as CS. A key issue is whether US-retrieval induces the process of memory reconsolidation differently from CS-retrieval.

Converging evidence using rodents and human subjects reveals that noradrenergic signaling is critically involved in CS-retrieval induced memory reconsolidation of fear conditioning. Systemic or intra-LA injection of β-AR antagonist after retrieval reduces fear memory in rats (Debiec and Ledoux, 2004; Muravieva and Alberini, 2010); disruption of noradrenergic signaling during reconsolidation process reduces long-term emotional memory in healthy humans (Lonergan et al., 2013; Kroes et al., 2014). The downstream signals of β-AR, such as PKA (Tronson et al., 2006), MAPK (Duvarci et al., 2005) and CREB (Tronson et al., 2012), are involved in fear memory reconsolidation. Besides, norepinephrine infusion in the basolateral amygdala (BLA) enhances extinction of contextual fear conditioning (Berlau and McGaugh, 2006; Roozendaal and McGaugh, 2011). Furthermore, β-adrenoreceptor stimulation is involved in other memories, such as social reward-related memory (Achterberg et al., 2012), cocaine-associated memory (Achterberg et al., 2012; Otis et al., 2013), and object recognition memory (Roozendaal et al., 2008; Liu et al., 2015). The above studies have suggested that β-AR and its downstream signaling pathways are involved in CS-induced memory reconsolidation. However, whether β-AR dependent signaling involved in US triggered memory reconsolidation is unknown.

Here, we addressed these issues by using a sequential conditioning including two different sounds associated with footshock (FS) in the training session. Then the effects of β-AR blockade on US-retrieval or CS-retrieval triggered memory reconsolidation were tested, and CREB activation induced by US-retrieval or CS-retrieval in amygdala and hippocampus was examined. Our data revealed that β-adrenergic signaling mediates US triggered memory reconsolidation of fear conditioning, and US-retrieval might activate more memory traces than CS-retrieval does.

## Materials and Methods

### Animals

Seven-week-old male C57BL/6J mice were purchased from Slaccas Lab Animal Ltd, Shanghai, China, weighing about 22 g. Adult male C57BL/6J mice (22–26 g) aged from 8–10 weeks old were used in all studies. Mice were housed in plastic Nalgene cages in a humidity- and temperature-controlled room and maintained on a reversed 12-h light/dark cycle (Light from 20:00–8:00). Food and water were provided *ad libitum* throughout the experiment. All animal treatments were strictly in accordance with the National Institutes of Health Guide for the Care and Use of Laboratory Animals, and were approved by Animal Care and Use Committee of Shanghai Medical College of Fudan University.

### Drug Treatment

(+)-Propranolol HCL and Betaxolol purchased from Sigma (Sigma-Aldrich, USA) were dissolved in saline. The drugs were intraperitoneally injected 5 min after memory retrieval with a dose of 10 mg/kg (Propranolol and betaxolol). Controls received an equivalent volume of saline (Ns: 4 ml/kg).

### Behavioral Apparatus and Stimuli

All procedures were conducted in a custom-made conditioning chamber (Med Associates Inc. #MED-VFC-SCT-M, height × width × length: 20 × 20 × 20 cm^3^). The walls of the chamber were constructed of stainless-steel bars, and the floor was a standard conditioning chamber grid rods used for delivering FS. The conditioning chamber was ventilated acoustic isolation box. A diffuse light illuminated the chamber during the procedures. Behavior was recorded using a camera on the door of each isolation box.

The CS (conditioned stimuli) were two distinct sounds (CS-A: 2800 kHz tone, 50 ms duration, 85 dB, 50 ms rise time and CS-B: noise, 60 dB, 50 ms rise time, 20-s each). The US (ununconditioned stimulus) was a 1.0-s, 0.25-mA or 0.5-mA electric FS delivered through the grid floor.

### Behavioral Procedures

#### Habituation to Context (Days 1–3)

Mice were placed in the conditioning chamber and habituated for 30 min on three consecutive days.

#### Conditioning (Day 4)

In the conditioning context, three CS-A/FS pairings with 30-s intertrial interval followed by three CS-B/FS pairings were given after an adaptation period of 180 s. The intersessional interval between conditioning of CS-A/FS and CS-B/FS was 180 s. The FS was delivered at the end of each sound and co-terminated with sound. Thirty seconds after last paired conditioning, the mice were taken out of the chamber and returned to their homecage.

#### Memory Retrieval (Day 5)

A single FS with the same intensity and duration as conditioning was presented 180 s after exposure to conditioning context as US-retrieval. With an additional 30-s in this context, the mice were taken out and returned to their homecage. In this study, the US was presented in the same context of training, where the animals had already well adapted. No novel information were introduced during the US-retrieval process. The context and US were only part of previous associative memory. Then US-retrieval might not form a new memory. The mice of control group were kept at homecage or only exposed to the context for 210 s without FS. A different cohort of mice were introduced to the context and presented with a 20-s sound (CS-A or CS-B) as CS-retrieval. Similar to US-retrieval, CS-retrieval did not produce new memory. Then β-blocker was injected intraperitoneally 5 min after the exposure to FS or sound or the conditioning context.

#### Short-Term Memory (STM) Retention Tests (Day 5)

One hour after the retrieval session, mice were placed in a novel context and tested for STM retention. Four 20-s CS-As were presented, followed by four 20-s CS-Bs with a 180-s intersessional interval. The intertrial interval was 30 s.

#### Long-Term Memory (LTM) Retention Tests (Day 6)

Twenty four hours after retrieval, mice were given a LTM retention test. Four CS-As and four CS-Bs were presented sequentially in a novel context as STM test.

### Immunohistochemistry

Mice were anesthetized by chloral hydrate (10%) and perfused intracardiacally with 0.9% saline first, then with 4% paraformaldehyde in 0.1 M Na_2_HPO_4_/NaH_2_PO_4_ buffer (pH = 7.5) containing 1 mM NaF. Brains were quickly removed, po M NaF for at least 24 h. Brain slices were sectioned into 30 μm by a vibratome (Leica). Floating sections were incubated in primary antibody against pCREB (Abcam, phospho S133; 1:500) at 4°C overnight. After rinsing in PBS/NaF, sections were incubated for 2 h with the biotinylated anti-rabbit IgG (1:200). Sections were rinsed and incubated at room temperature for 40 min in avidin-biotin-complex solution (ABC Solution; Vector Laboratories) in PBS. The peroxidase reaction was visualized in 0.01 M PBS containing 0.025% diaminobenzidine tetrahydrochloride and 0.03% H_2_O_2_. The quantification of pCREB-positive cells was carried out at 10× magnification or 20× magnification. At least 3 serial sections were digitized by using an image analysis system (Spot Advanced 4.1.2, Diagnostic instruments, Inc.) and analyzed by Image-Pro Plus. Structures were defined according to the Franklin and Paxinos atlas (Paxinos and Franklin, 2004). Labeled cells above a same threshold determined from control animals were counted (Brami-Cherrier et al., 2005). The vehicle group without memory retrieval was treated as control. Data in the graphs are presented as the mean ± SEM of positive cell counts per mm^2^.

### Data Analysis

Data were analyzed by using two-way repeated measures ANOVA or two-way ANOVA with treatment as between-subject factors and session/trial as within-subject factor. Significant effects were analyzed using a single interaction and a *post hoc* Tukey’s test for the behavioral analysis or Bonferroni’s test for IHC analyses.

## Results

### β-AR Blockade after US-Retrieval Impaired Memory Reconsolidation of Conditioned Fear Associated with Both CS-A and CS-B

**Figure 1:**
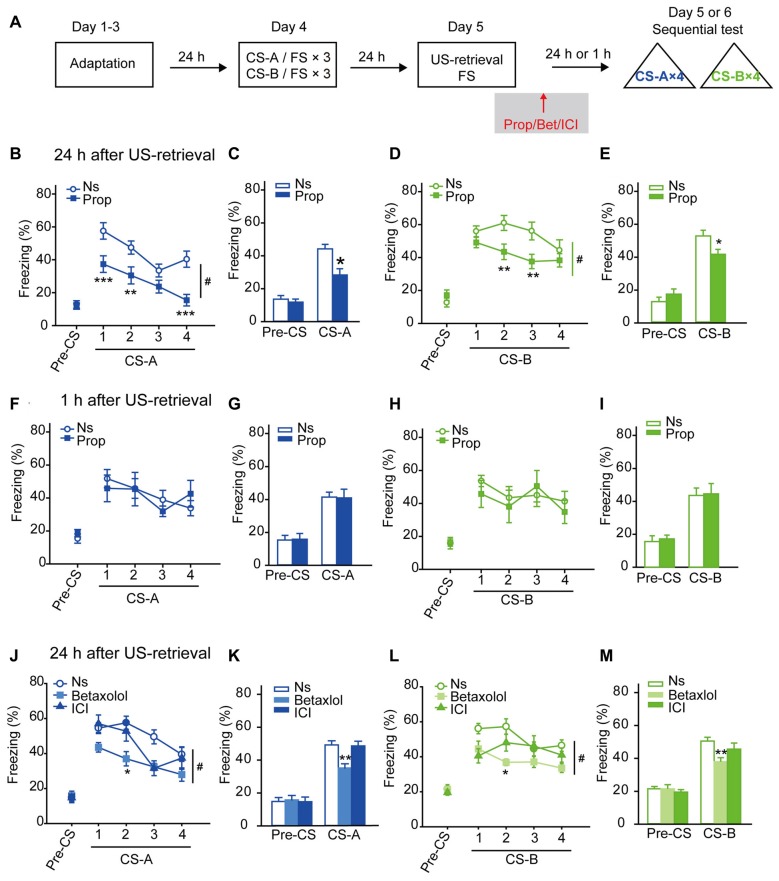
Long-term memory (LTM) of fear conditioning was disrupted by administration of β-blocker after unconditioned stimulus (US)-retrieval. **(A)** Schematic of the main experimental design. Animals were trained in a sequential fear conditioning paradigm with three pairs of conditioned stimulus (CS) (CS)-A/US followed by three pairs of CS-B/US (FS: 0.25 mA). The next day, a single footshock (FS; 0.25 mA) was given as US-retrieval followed by β-blocker treatment (propranolol, betaxolol or ICI118,551). **(B–E)** Twenty-four hours after propranolol injection, LTM was tested as the freezing behavior in response to both CS-A and CS-B. **(B,D)** Curves of response to CS showed as the percentage time of freezing during each CS-A and CS-B. **(C,E)** Freezing to CS-A or CS-B in average. *n* = 16 for Prop group; *n* = 17 for Ns group. **p < 0.01 vs. Ns group; ***p < 0.001 vs. Ns group; ^#^*p* < 0.05 between indicated group. **(F–I)** One hour after propranolol treatment, mice were tested for short-term memory (STM) with both CS-A and CS-B. **(F,H)** Curves of response to CS showed as the percentage time of freezing during each CS. **(G,I)** Freezing to CS-A or CS-B in average. *n* = 8 for Prop group; *n* = 8 for Ns group. **(J–M)** Twenty-four hours after treatment of betaxolol or ICI118,551, mice were tested for fear memory in response to both CS-A and CS-B. **(J,L)** Curves of response to CS showed as the percentage time of freezing during each CS. **(K,M)** Freezing to CS-A or CS-B in average. *n* = 10 for Betaxolol group; *n* = 13 for ICI group; *n* = 18 for Ns group. *p < 0.05 vs. Ns treated group; **p < 0.01 vs. Ns treated group; ^#^*p* < 0.05 between indicated group.

After a three-day adaptation in the context where FS was delivered, the FS was conditioned with two distinct auditory stimuli sequentially: three pairs of CS-A/FS and followed by three pairs of CS-B/FS. On the following day, mice were exposed to a single FS as US-retrieval. To test whether β-AR is involved in US-retrieval induced reconsolidation of fear memory, propranolol was treated immediately after FS presentation (Figure 1A). Then memory retrieval evoked LTM was tested 24 h later as the percentage time of freezing in corresponding to each sound. With a strong conditioning procedure (FS: 0.5 mA), the propranolol treated group showed decreased trend of freezing levels to CS-A and CS-B compared with the saline treated group, with no treatment-by-trial interaction (Supplementary Figures S1A,B,D, *F*_treatment × trial (4,80)_ = 1.630, *p* = 0.175 for CS-A; *F*_treatment × trial (4,80)_ = 2.142, *p* = 0.083 for CS-B, two-way RM ANOVA). In average of the freezing levels to four CS, ANOVA revealed a significantly inhibitory effect of propranolol treatment on both CS-A and CS-B evoked freezing behavior and a treatment-by-session test interaction (Supplementary Figures S1C,E, *F*_treatment × session (1,20)_ = 7.947, *p* = 0.011 for CS-A; *F*_treatment × session (1,20)_ = 5.213, *p* = 0.033 for CS-B, two-way RM ANOVA). With a mild conditioning procedure (FS: 0.25 mA), propranolol treatment significantly decreased freezing levels in response to CS-A and CS-B, (Figures 1B,D, *F*_treatment × trial (4,124)_ = 3.156, *p* = 0.016 for CS-A; *F*_treatment × trial (4,124)_ = 3.281, *p* = 0.014 for CS-B, two-way RM ANOVA). In average, ANOVA revealed a significant suppression of propranolol on freezing in response to both CS-A and CS-B and a treatment-by-session interaction (Figures 1C,E, *F*_treatment × session (1,31)_ = 6.825, *p* = 0.014 for CS-A; *F*_treatment × session (1,31)_ = 5.950, *p* = 0.021 for CS-B, two-way RM ANOVA). *Post hoc* analysis confirmed that animals froze less to both sounds with treatment of propranolol after FS presentation, suggesting β-AR antagonism treatment after US-retrieval impaired LTM of fear conditioning. Then the FS intensity of 0.25 mA was used in the training session for all the other procedures. In addition, we found β-blocker treatment significantly decreased freezing levels in response to CS in the first and second trials in the memory retention test 24 h after memory retrieval (Figure 1B CS-A: *F*_(1,31)_ = 10.211, *p* = 0.003; Figure 1D CS-B: *F*_(1,31)_ = 6.215, *p* = 0.019; Figure 1J CS-A: *F*_(2,38)_ = 5.740, *p* = 0.007; Figure 1L CS-B: *F*_(1,38)_ = 6.609, *p* = 0.003, one-way ANOVA), suggesting this memory impairment should not be due to extinction.

Then the effects of propranolol treatment after US-retrieval on STM were tested. One day after the sequential fear conditioning paradigm, the mice were exposed to one FS followed by either propranolol or saline treatment (i.p.). Fear memory was assessed 1 h after FS presentation (Figure 1A). Freezing percentage in response to exposure to each CS-A and CS-B was assessed, and no inhibition by propranolol treatment was detected (Figures 1F,H *F*_treatment × trial (4,56)_ = 0.723, *p* = 0.580 for CS-A; *F*_treatment × trial (4,56)_ = 0.517, *p* = 0.723 for CS-B, two-way RM ANOVA). In average, freezing levels were not suppressed by propranolol in STM retention test (Figures 1G,I *F*_treatment × session (1,14)_ = 0.033, *p* = 0.858 for CS-A; *F*_treatment × session (1,14)_ = 0.006, *p* = 0.940 for CS-B, two-way RM ANOVA), suggesting propranolol treatment after US-retrieval does not impair STM of fear conditioning.

The effects of propranolol on conditioned fear memory without memory retrieval were also tested. Two days after the sequential fear conditioning paradigm, memory retention were tested without FS or sound presentation. Animals received either propranolol or saline (i.p.) at homecage or right after exposure to the conditioning context 24 h after training. Freezing levels to each CS-A and CS-B were tested one day after propranolol injection (Supplementary Figure S2A). No deficits in fear memory were observed by propranolol treated at homecage (Supplementary Figures S2B–E), *F*_treatment × trial (4,60)_ = 1.059, *p* = 0.385 for CS-A; *F*_treatment × trial (4,60)_ = 0.405, *p* = 0.804 for CS-B; in average, *F*_treatment × session (1,15)_ = 1.360, *p* = 0.262 for CS-A; *F*_treatment × session (1,15)_ = 0.254, *p* = 0.621 for CS-B, two-way RM ANOVA), or after contextual exposure (Supplementary Figures S2F–I), *F*_treatment × trial (4,72)_ = 0.352, *p* = 0.842 for CS-A; *F*_treatment × trial (4,72)_ = 0.522, *p* = 0.720 for CS-B; in average, *F*_treatment × session (1,18)_ = 0.072, *p* = 0.791 for CS-A; *F*_treatment × session (1,18)_ = 0.529, *p* = 0.477 for CS-B, two-way RM ANOVA).

The results above suggest that β-AR is required for memory reconsolidation of conditioned fear memory induced by US-retrieval, and β-AR blockade afterward might disrupt the associations of multiple CS with US.

Betaxolol (selective β1-AR antagonist) or ICI 118,551 (selective β2-AR antagonist) was injected 5 min after US-retrieval and 24 h later memory retention tests were performed (Figure 1A). Treatment of betaxolol, but not ICI 118,551, significantly decreased freezing levels in response to CS-A and CS-B (Figures 1J,L, *F*_treatment × trial (8,152)_ = 2.500, *p* = 0.014 for CS-A; *F*_treatment × trial (8,152)_ = 2.313, *p* = 0.023 for CS-B, two-way RM ANOVA). In average, ANOVA revealed a significantly suppressive effects of betaxolol on freezing in response to both CS-A and CS-B and a treatment-by-session test interaction (Figures 1K,M, *F*_treatment × session (2,38)_ = 4.499, *p* = 0.018 for CS-A; *F*_treatment × session (2,38)_ = 4.082, *p* = 0.025 for CS-B, two-way RM ANOVA). *Post hoc* analyses confirmed that animals treated with betaxolol froze less to CS-A and CS-B. The results indicate that β1-AR might be involved in fear memory reconsolidation induced by US-retrieval.

### β-AR Blockade Following CS-Retrieval Selectively Impaired Fear Memory Reconsolidation Evoked by the Same CS

**Figure 2:**
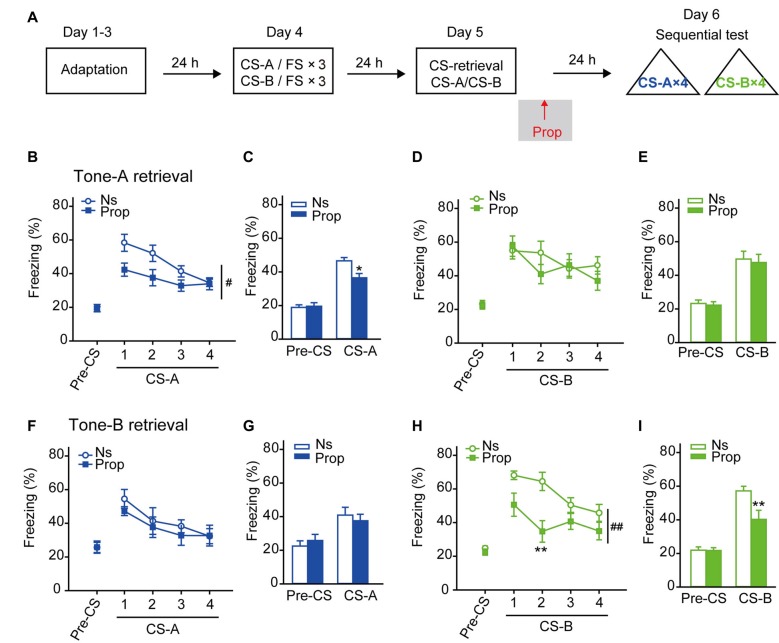
Propranolol treatment after CS-retrieval disrupted fear memory reconsolidation selectively in response to the same CS. **(A)** Schematic of the main experimental design. Twenty-four hours after the sequential fear conditioning paradigm (FS: 0.25 mA), propranolol was treated after CS-A or CS-B induced memory retrieval. Twenty-four hours after CS-retrieval, mice were tested for LTM to both CS-A and CS-B. **(B–E)** Selective impairment of fear memory reconsolidation to CS-A by propranolol treated after CS-A retrieval. **(B,D)** Curves of response to CS showed as the percentage time of freezing during each CS. **(C,E)** Freezing to CS-A or CS-B in average. *n* = 12 for Propranolol group; *n* = 11 for Ns group. *p < 0.05 vs. Ns treated group; ^#^*p* < 0.05 between indicated group. **(F–I)** Selective impairment of fear memory reconsolidation to CS-B by propranolol treated after CS-B retrieval. **(F,H)** Curves of response to CS showed as the percentage time of freezing during each CS. **(G,I)** Freezing to CS-A or CS-B in average. *n* = 8 for each group. **p < 0.01 vs. Ns treated group; ^##^*p* < 0.01 between indicated group.

Next, we proceeded to determine the effects of β-AR blockade on cued fear memory reconsolidation. The same training paradigm was used, and then propranolol was treated after CS-A or CS-B presentation (Figure 2A). The freezing behavior to each CS-A and CS-B was tested 24 h after CS-retrieval. In CS-A retrieval paradigm, propranolol treatment only inhibited freezing in response to CS-A, but not to CS-B (Figures 2B,D, *F*_treatment × trial (4,84)_ = 2.537, *p* = 0.046 for CS-A; *F*_treatment × trial (4,84)_ = 1.693, *p* = 0.159 for CS-B, two-way RM ANOVA). In average of freezing levels responding to each 4 CS, ANOVA revealed a significantly decreased effect of propranolol on freezing response to CS-A, but not to CS-B (Figures 2C,E, *F*_treatment × session (1,21)_ = 10.005, *p* = 0.005 for CS-A; *F*_treatment × session (1,21)_ = 0.023, *p* = 0.881 for CS-B, two-way RM ANOVA). Similarly, in CS-B retrieval paradigm, propranolol only inhibited freezing in response to CS-B, but not to CS-A (Figures 2F–I, *F*_treatment × trial (4,56)_ = 0.553, *p* = 0.698 for CS-A; *F*_treatment × trial (4,56)_ = 4.725, *p* = 0.002 for CS-B; in average *F*_treatment × session (1,14)_ = 1.268, *p* = 0.279 for CS-A; *F*_treatment × session (1,14)_ = 7.656, *p* = 0.015 for CS-B, two-way RM ANOVA). This inhibitory effect of propranolol on fear memory reconsolidation is consistent with previous reports that β-AR blockade after CS induced memory retrieval impairs the reconsolidation of cued fear memory (Debiec and Ledoux, 2004). Furthermore, our result showed that propranolol treatment after CS-retrieval selectively decreased freezing behavior induced by the same CS in the subsequent memory test, indicating that the retrieval by a particular CS leads to memory reconsolidation process selectively. Thus, when the stimuli are separated in time during conditioning, memory retrieved by the CS is discrete and reconsolidates separately.

### β-AR-Dependent CREB Activation Induced by US-Retrieval was Distinct from that Induced by CS-Retrieval

**Figure 3:**
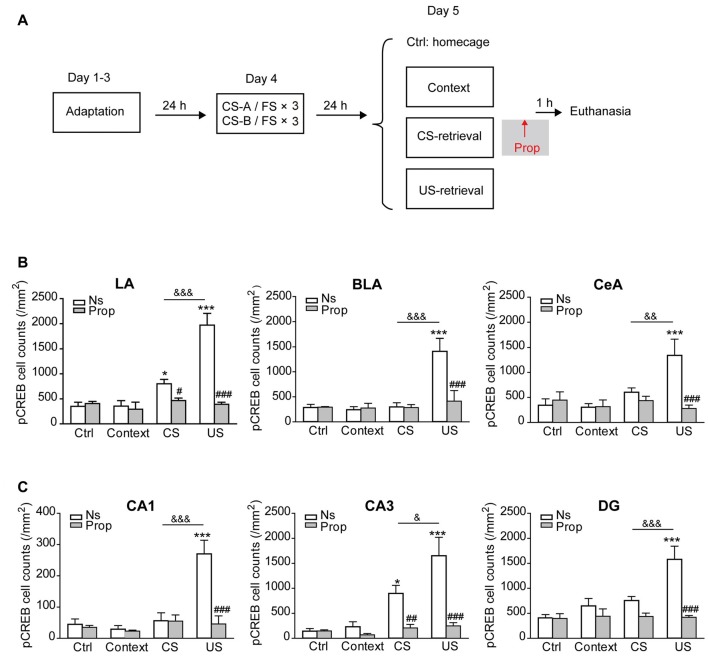
CREB was activated in the amygdala and hippocampus by memory retrieval, which was suppressed by propranolol treatment. **(A)** The behavioral procedure for pCREB expression tests by IHC. **(B)** Quantification of pCREB immunopositive cells in the amygdala. *n* = 4–9. *p < 0.05, ***p < 0.001 vs. Ctrl-Ns group; ^#^*p* < 0.05, ^###^*p* < 0.001 vs. CS-Ns or US-Ns group; ^&&^*p* < 0.01, ^&&&^*p* < 0.001 between indicated groups. **(C)** Quantification of pCREB immunopositive cells in the hippocampus. *n* = 4–9. *p < 0.05, ***p < 0.001 vs. Ctrl-Ns group; ^##^*p* < 0.01, ^###^*p* < 0.001 vs. CS-Ns or US-Ns group; ^&^*p* < 0.05, ^&&&^*p* < 0.001 between indicated groups.

The data above indicate that US-retrieval induced memory reconsolidation process is distinct from CS-retrieval, as propranolol treatment after US-retrieval impaired the association of the US with both CS, while the treatment after the retrieval of one CS only impaired the association of the US with the corresponding CS specifically. To explore whether US-retrieval will induce brain nuclei activation differently from CS-retrieval, mice were exposed to a single FS as a US-retrieval, or a sound as a CS-retrieval one day after the sequential fear conditioning paradigm. The levels of pCREB immunoreactivity in brain sections of saline-treated control group were used to determinate the threshold for pCREB-positive cell counts for all groups. Our data showed US-retrieval and CS-retrieval induced differential CREB activation in the amygdala and hippocampus in mice received sequential fear conditioning (Figure 3A).

First, the IHC data revealed that both US-retrieval and CS-retrieval could induce significant CREB activation in the amygdala, which could be suppressed by injection of propranolol (Figure 3B, Supplementary Figure S3). In detail, both US-retrieval and CS-retrieval increased pCREB positive cell number in the LA. Furthermore, US-retrieval significantly increased pCREB positive cells in the BLA and the central amygdala (CeA). Propranolol treatment immediately after memory retrieval reduced the pCREB levels to the baseline (LA: *F*_treatment × retrieval (3,44)_ = 19.750, *p* < 0.001; BLA: *F*_treatment × retrieval (3,44)_ = 5.370, *p* = 0.003; CeA: *F*_treatment × retrieval (3,44)_ = 5.500, *p* = 0.003, two-way ANOVA).

Next, the pCREB levels in the hippocampus were tested and analyzed after memory retrieval. As shown in Figure 3C, and Supplementary Figure S4, US-retrieval significantly increased pCREB positive cells in the CA1, CA3 and DG regions of the hippocampus, while CS-retrieval only increased CREB activation in the CA3. Propranolol inhibited CREB activation induced by both US-retrieval and CS-retrieval (CA1: *F*_treatment × retrieval (3,44)_ = 9.272, *p* < 0.001; CA3: *F*_treatment × retrieval (3,44)_ = 6.430, *p* < 0.001; DG: *F*_treatment × retrieval (3,44)_ = 7.204, *p* < 0.001, two-way ANOVA). The levels of CREB activation in the mPFC were also tested after memory retrieval, while no significant changes of pCREB were detected after CS-retrieval or US-retrieval (Supplementary Figure S5A PrL: *F*_treatment × retrieval (3,44)_ = 0.502, *p* = 0.683; IL: *F*_treatment × retrieval (3,44)_ = 0.720, *p* = 0.546). In addition, US-retrieval and CS-retrieval increased pCREB positive cell counts in the ventral part of secondary auditory cortex (AuV) and the medial geniculate nucleus (MGm), which was inhibited by propranolol treatment (Supplementary Figure S5B AuV: *F*_treatment × retrieval (3,44)_ = 4.399, *p* = 0.009; MGm: *F*_treatment × retrieval (3,44)_ = 3.451, *p* = 0.024).

Collectively, our results reveal that CS-retrieval induces activation of LA and CA3, while US-retrieval induces more brain nuclei activation in the amygdala and hippocampus, and US-retrieval induced activation is dependent on β-AR signaling.

## Discussion

The present results showed that administration of β-AR antagonism after US-retrieval disrupted fear memory reconsolidation in response to both CS, while β-AR blockade after CS-retrieval only selectively impaired fear memory reconsolidation evoked by the same CS. US-retrieval-induced fear memory reconsolidation was dependent on β-AR. Moreover, US-retrieval induced greater β-AR-dependent CREB activation in the amygdala and hippocampus than CS-retrieval did. We speculate that US and CS trigger differential reconsolidation processes and US presentation alone would render multiple CS-US associations susceptible to disruption.

Recent study has shown that two fear-conditioning events that occur within 6 h are coallocated to overlapping populations of neurons in the LA, and extinction of event2 memory by presenting CS2 decreases the fear memory related to CS1, indicating that memories links when occur closely (Rashid et al., 2016). However, study from Silva’ lab has shown lately that fear paired with one context is transferred to a second context when the two contexts are acquired within one day, but extinction training of the second context keeps the first contextual fear memory intact (Cai et al., 2016). Although memory encoding of two CS-US associations will go to the same neurons when two associations formed closely in time, it is still not clear whether impairment of one CS-US association will affect others or not. The organization of memory formation needs further investigation. Several recent studies have shown that memory is retrieved by the presentation of CS discretely and reconsolidated separately. When FS is associated with two distinct neutral events separately and only one event is used for memory retrieval, anisomycin, a protein synthesis inhibitor, injected in LA post-recall only inhibits this event induced freezing, but not another (Debiec et al., 2013). The human and animal studies from Lu’s lab have shown that either CS1 or CS2 extinction after exposure to CS1 only disrupts the reconsolidation of fear conditioning or drug memory in response to CS1 (Liu et al., 2014; Luo et al., 2015). In this study, we found that propranolol treatment after CS-retrieval only impaired the same CS associated fear memory reconsolidation in a sequential training paradigm, which was consistent with previous studies. In a majority of reconsolidation studies so far, memory is typically reactivated by a single CS presentation, yet, in real life, stimuli is always exposed and associated with different modalities. Then the question arises, how to largely suppress the fear memory in those patients with memory related disorder? Consistent with other studies (Díaz-Mataix et al., 2011; Liu et al., 2014), we found that US itself was a strong reminder (Milekic et al., 2006), which could activate more memory traces than a single CS does. When interventions, such as β-blockers which are used for treatment of hypertension in clinic, were introduced within the time window of labile state of memory induced by US-retrieval, fear memory reconsolidation of all CS-US associations was impaired. However, in this study, treatment after memory retrieval decreased freezing levels in the memory retention test, but animals still showed freezing, suggesting that β-blockade after memory retrieval impaired memory reconsolidation, but did not erase the memory completely.

A large body of evidence implicates amygdala as a key component of the neural system involved in memory acquisition and storage of fear conditioning, especially when an auditory cue is used. The anatomical convergence of CS and US information in the LA leads to the view that associative learning is mediated by synaptic plasticity in this region (Goosens and Maren, 2002). Overexpression of the inducible cAMP early repressor or the dominant-negative mCREB, within the LA impaired reconsolidation of auditory fear memories (Tronson et al., 2012). Elevated CREB expression or its activation in the LA was critical for CS-retrieval induced fear memory reconsolidation (Tronson et al., 2012; Kim et al., 2014). The elevated levels of CREB phosphorylation in the LA was induced by cue presentation in a β-AR-dependent manner (Johansen et al., 2011). Consistently, our data showed CS-retrieval increased CREB activity in the LA. More importantly, our results showed that US-retrieval induced greater CREB activation in the LA than CS-retrieval did. The two main auditory brain regions, the MGm and AuV send axonal projections to the LA (Kwon et al., 2014). In this study, the AuV and MGm were both activated by US or CS induced memory retrieval, indicating that US itself can activated auditory brain regions after paired with sounds. Besides, US-retrieval significantly increased CREB activity in other subnucleus of amygdala, such as BLA and CeA. Studies suggest that synaptic plasticity mechanisms in the CeA are critical for the acquisition and consolidation of fear memories, which is another site for the convergence of CS and US information (Paré et al., 2004). The basolateral complex also contributes to storage and expression of fear memory (Maren, 1999, 2001a). In this study, after fear memory acquired and consolidated, US exposure fully activated the amygdala, while CS exposure only partially activated the amygdala. The roles of the hippocampus in Pavlovian fear conditioning have also been extensively studied. Many subsequent studies have found that the hippocampal lesions lead to impairments in contextual conditioning (Kim et al., 1993; Antoniadis and McDonald, 2000). Based on these results, the wildly held view is that the hippocampus is always required for fear conditioning to contexts, but never cues. However, some studies showed that the hippocampal lesions produced reliable deficits in freezing to the auditory CS in memory retention test (Maren and Holt, 2004). As to memory reconsolidation, the activation of the transcription factor CREB increased greatly in the CA1 and CA3, when compared to the non-associative training group (Mamiya et al., 2009). In our study, results showed that mild CREB activation was detected in the CA3 after CS-retrieval, and stronger CREB activation was found in the CA3, CA1 and DG in US-retrieval group. Taken together, our results indicate that the amygdala and hippocampus are critically involved in US-retrieval induced memory reconsolidation of fear conditioning and US-retrieval triggers more memory traces activation in these brain nuclei than CS-retrieval does. Moreover, the widespread “network of memory” for the CS and for the US appears to include also the sensory system (e.g., thalamus and cortex; Ku et al., 2015).

In conclusion, our findings suggest that US retrieval activates more memory traces than CS retrieval does. US-retrieval induces memory reconsolidation of multiple associations, while CS induces memory reconsolidation discretely and selectively. Furthermore, US triggered fear memory reconsolidation is mediated by β-AR activation. Memories of aversive events often link with multiple cues and inhibiting response to all cues by exposing them separately is impractical, and there are viable translational methods to adapt a “US” presentation paradigm for the treatment of fear/anxiety for potential application in humans with VR. The US-retrieval combined with β-AR antagonism could be a potential strategy for the treatment of memory disorders, such as substance addiction or post-traumatic stress disorders. The neuronal circuitry underlying memory reconsolidation triggered by US-retrieval or CS-retrieval should be studied in our future research (Zhu et al., 2017).

## Author Contributions

XL and LM designed the research. BH and XL analyzed the data and wrote the article. BH, HZ and YZ performed the research and analyzed the data.

## Conflict of Interest Statement

The authors declare that the research was conducted in the absence of any commercial or financial relationships that could be construed as a potential conflict of interest.

## Supplementary Material

FIGURE S1 Long-term memory (LTM) of stronger fear conditioning was disrupted by administration of propranolol after unconditioned stimulus (US)-retrieval. **(A)** Schematic of the main experimental design. Animals were trained in a sequential fear conditioning paradigm with three pairs of CS-A/US followed by three pairs of CS-B/US (FS: 0.5 mA). The next day, a single foot shock (FS; 0.5 mA) was given as US-retrieval followed by propranolol treatment. **(B–E)** Twenty-four hours after US-retrieval, LTM was tested as the freezing behavior in response to both CS-A and CS-B. **(B,D)** Curves of response to conditioned stimulus (CS) showed as the percentage time of freezing during each CS. **(C,E)** Freezing to CS-A or CS-B in average. *n* = 11 for Prop group; *n* = 11 for Ns group. **p* < 0.05 vs. Ns group.

FIGURE S2 Memory of fear conditioning was not affected by administration of propranolol without memory retrieval. **(A)** Schematic of the main experimental design. Twenty-four hours after the sequential fear conditioning paradigm (FS: 0.25 mA), propranolol was treated without memory retrieval. **(B–E)** Twenty-four hours after drug treatment at homecage, mice were tested for fear memory in response to both CS-A and CS-B. **(B,D)** Curves of response to CS showed as the percentage time of freezing during each CS. **(C,E)** Freezing to CS-A or CS-B in average. *n* = 9 for Prop group; *n* = 8 for Ns group.** (F–I)** Twenty-four hours after context exposure, mice were tested for fear memory in response to both CS-A and CS-B. **(F,H)** Curves of response to CS showed as the percentage time of freezing during each CS. **(G,I)** Freezing to CS-A or CS-B in average. *n* = 12 for Prop group; *n* = 8 for Ns group.

FIGURE S3 Representative images of immunohistochemical staining for pCREB in LA, basolateral amygdala (BLA) and central amygdala (CeA) 30 min after memory retrieval followed by propranolol treatment. Scale bar: 200 μm.

FIGURE S4 Representative images of immunohistochemical staining for pCREB in CA1, CA3 and DG 30 min after memory retrieval followed by propranolol treatment. Scale bar: 200 μm.

FIGURE S5 CREB was activated in the AuV and MGm by memory retrieval, but not mPFC. **(A)** Quantification of pCREB immunopositive cells in the PrL and IL. *n* = 4–9. **(B)** Quantification of pCREB immunopositive cells in the AuV and the MGm. *n* = 4–9. **p* < 0.05, ***p* < 0.01, ****p* < 0.001 vs. Ctrl-Ns group; ^##^*p* < 0.01, ^###^*p* < 0.001 vs. CS-Ns or US-Ns group. (PrL) prelimbic cortex, (IL) infralimbic cortex, (AuV) ventral part of secondary auditory cortex, (MGm) medial geniculate nucleus.
