# Supplementary figures and images for: Unconditioned- and Conditioned- Stimuli Induce Differential Memory Reconsolidation and β-AR-Dependent CREB Activation
Source: Front Neural Circuits. 2017 Aug 10;11:53. doi: 10.3389/fncir.2017.00053 (PMC5554378; doi:10.3389/fncir.2017.00053)

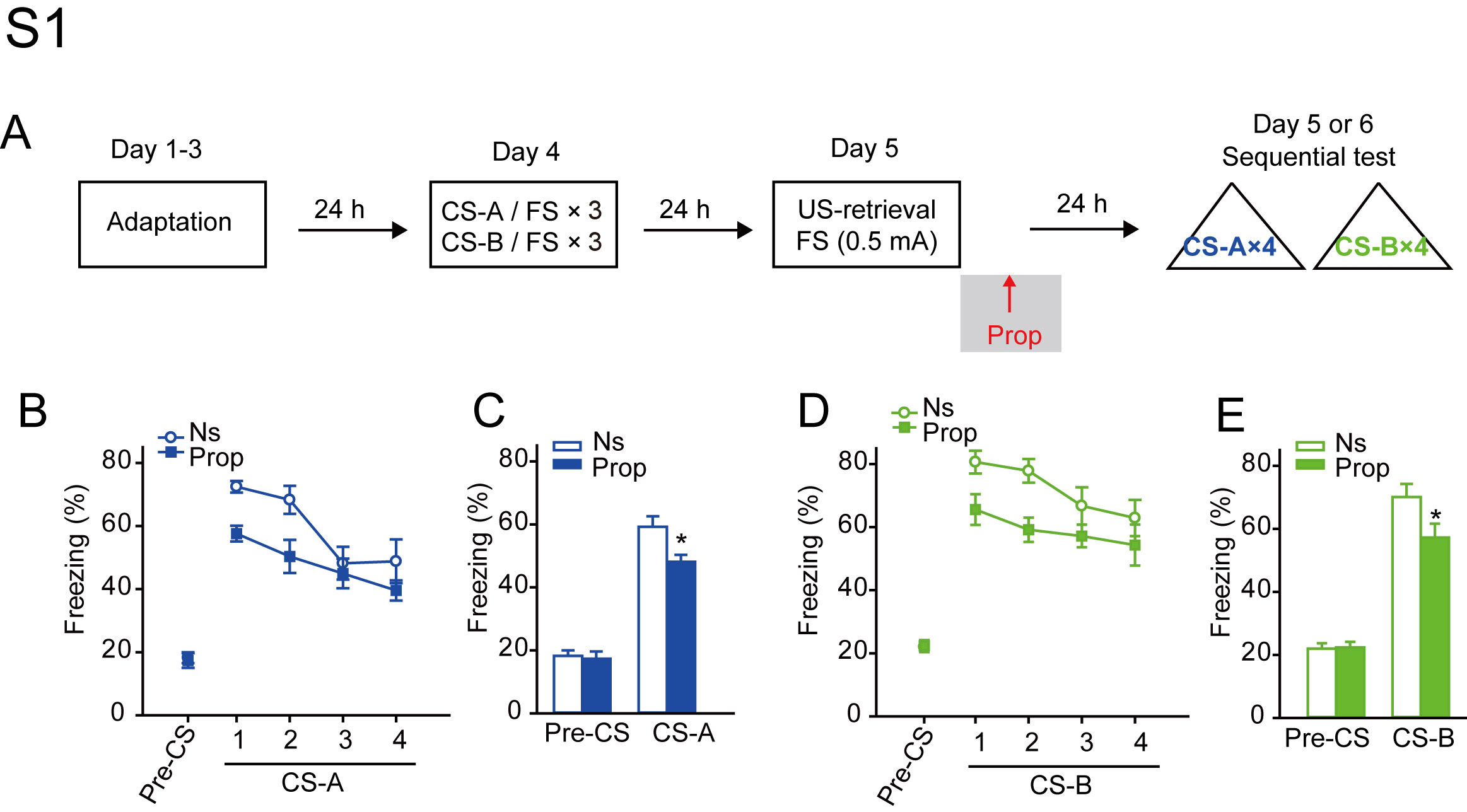

Supplement: FIGURE S1 — Long-term memory (LTM) of stronger fear conditioning was disrupted by administration of propranolol after unconditioned stimulus (US)-retrieval. (A) Schematic of the main experimental design. Animals were trained in a sequential fear conditioning paradigm with three pairs of CS-A/US followed by three pairs of CS-B/US (FS: 0.5 mA). The next day, a single foot shock (FS; 0.5 mA) was given as US-retrieval followed by propranolol treatment. (B–E) Twenty-four hours after US-retrieval, LTM was tested as the freezing behavior in response to both CS-A and CS-B. (B,D) Curves of response to conditioned stimulus (CS) showed as the percentage time of freezing during each CS. (C,E) Freezing to CS-A or CS-B in average. n = 11 for Prop group; n = 11 for Ns group. *p < 0.05 vs. Ns group. [file Image_1.jpeg]

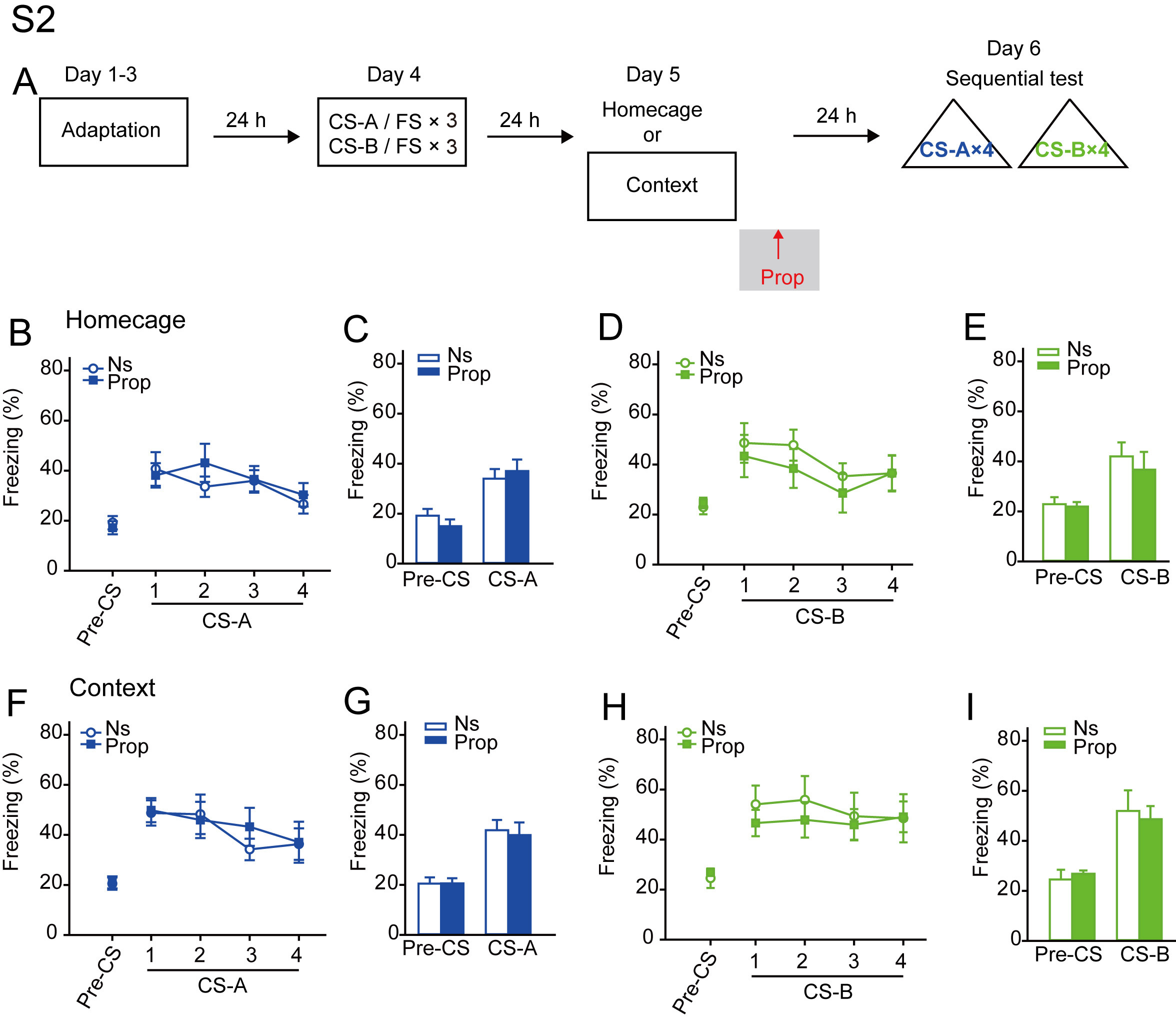

Supplement: FIGURE S2 — Memory of fear conditioning was not affected by administration of propranolol without memory retrieval. (A) Schematic of the main experimental design. Twenty-four hours after the sequential fear conditioning paradigm (FS: 0.25 mA), propranolol was treated without memory retrieval. (B–E) Twenty-four hours after drug treatment at homecage, mice were tested for fear memory in response to both CS-A and CS-B. (B,D) Curves of response to CS showed as the percentage time of freezing during each CS. (C,E) Freezing to CS-A or CS-B in average. n = 9 for Prop group; n = 8 for Ns group. (F–I) Twenty-four hours after context exposure, mice were tested for fear memory in response to both CS-A and CS-B. (F,H) Curves of response to CS showed as the percentage time of freezing during each CS. (G,I) Freezing to CS-A or CS-B in average. n = 12 for Prop group; n = 8 for Ns group. [file Image_2.jpeg]

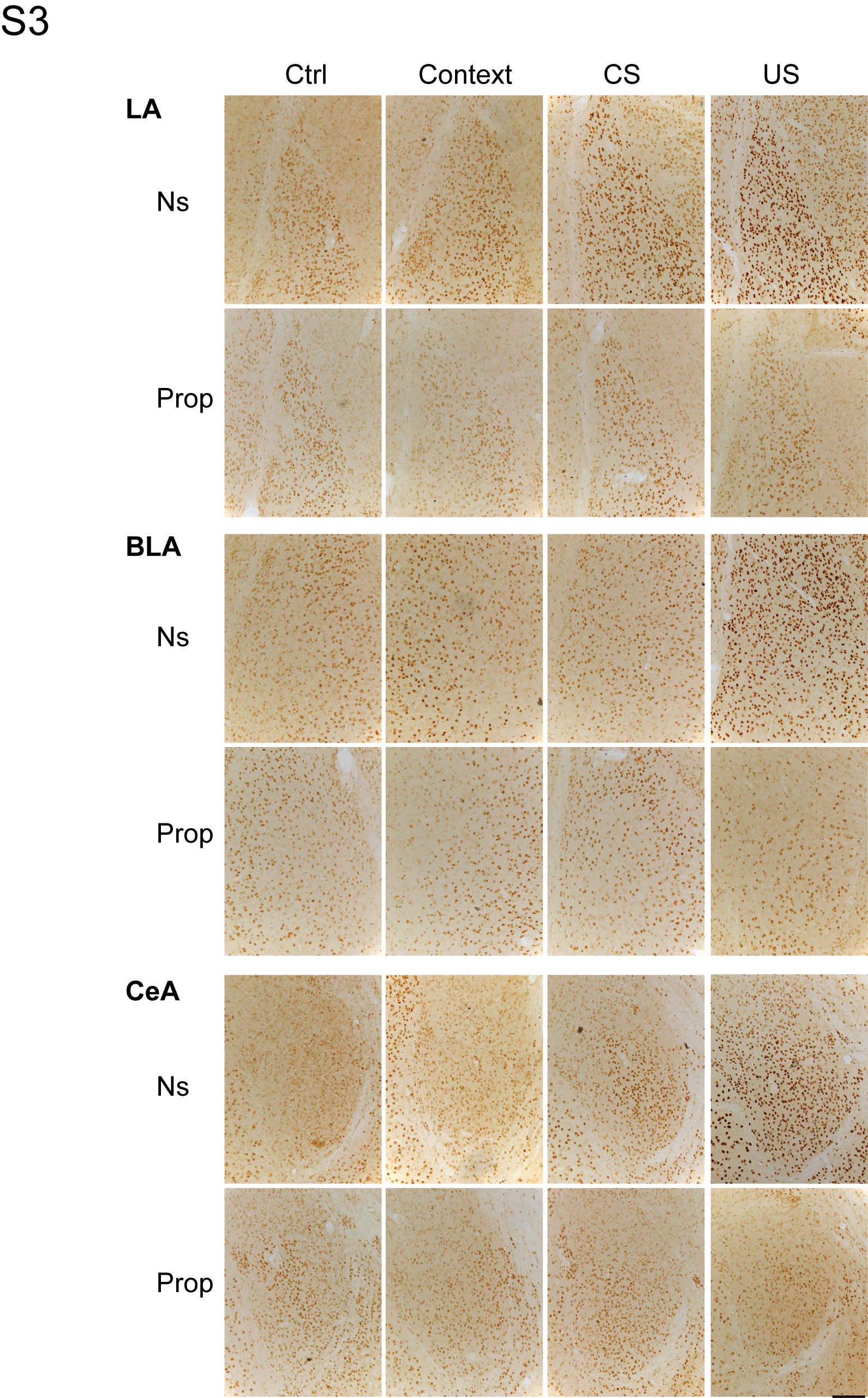

Supplement: FIGURE S3 — Representative images of immunohistochemical staining for pCREB in LA, basolateral amygdala (BLA) and central amygdala (CeA) 30 min after memory retrieval followed by propranolol treatment. Scale bar: 200 μm. [file Image_3.jpg]

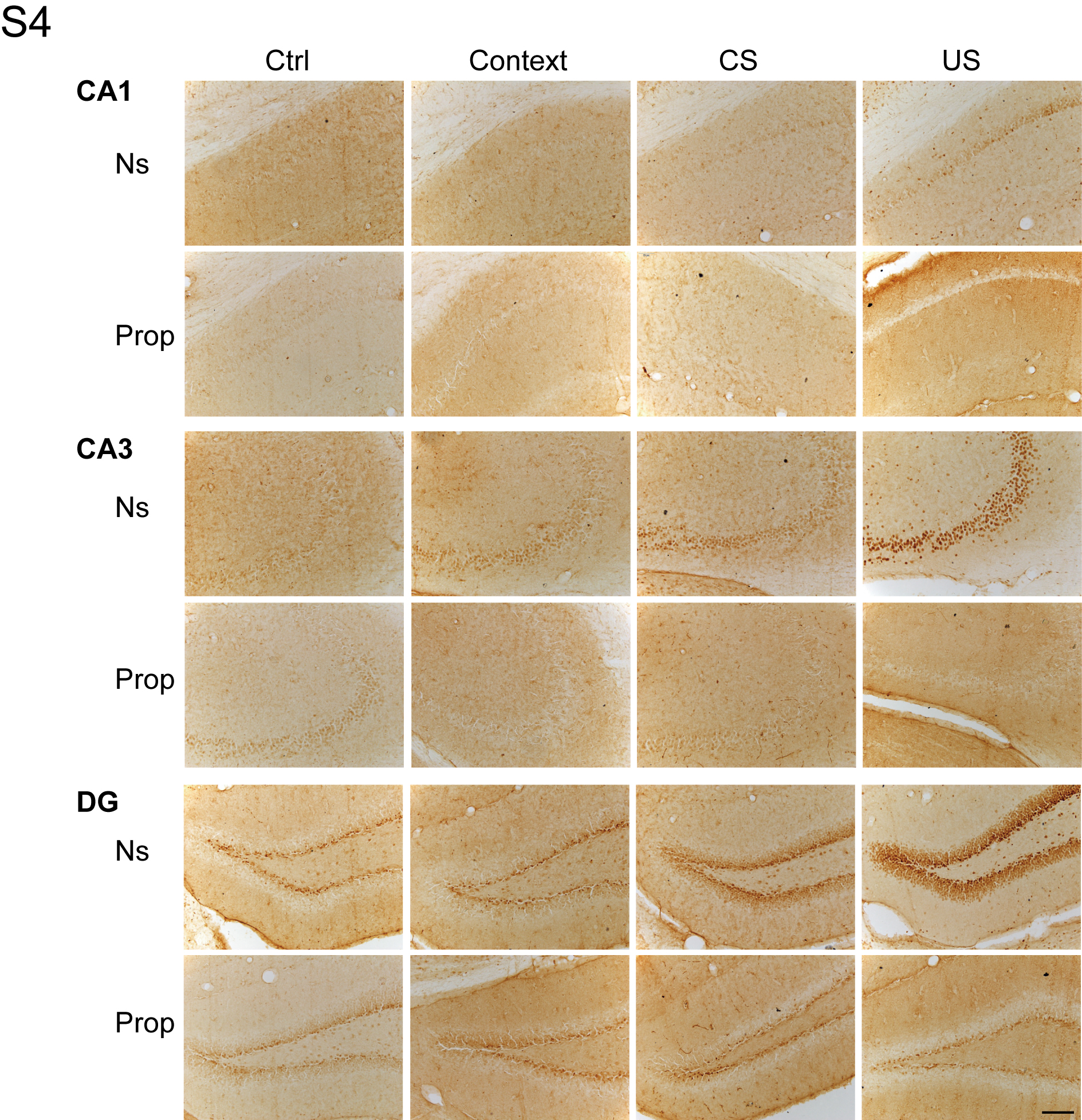

Supplement: FIGURE S4 — Representative images of immunohistochemical staining for pCREB in CA1, CA3 and DG 30 min after memory retrieval followed by propranolol treatment. Scale bar: 200 μm. [file Image_4.jpg]

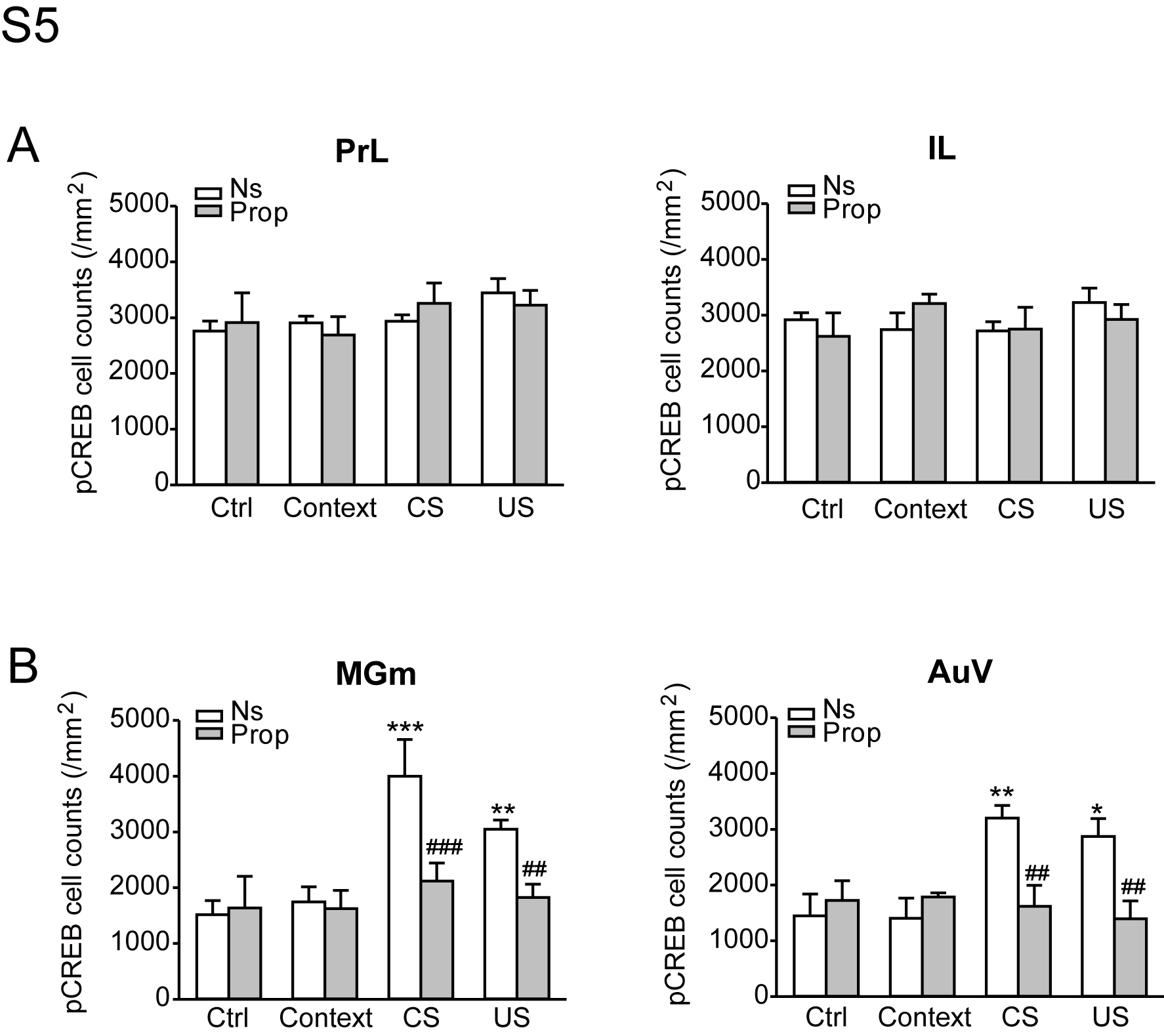

Supplement: FIGURE S5 — CREB was activated in the AuV and MGm by memory retrieval, but not mPFC. (A) Quantification of pCREB immunopositive cells in the PrL and IL. n = 4–9. (B) Quantification of pCREB immunopositive cells in the AuV and the MGm. n = 4–9. *p < 0.05, **p < 0.01, ***p < 0.001 vs. Ctrl-Ns group; ##p < 0.01, ###p < 0.001 vs. CS-Ns or US-Ns group. (PrL) prelimbic cortex, (IL) infralimbic cortex, (AuV) ventral part of secondary auditory cortex, (MGm) medial geniculate nucleus. [file Image_5.jpg]
